# Expression and distribution of activin-follistatin-inhibin axis in the urinary bladder

**DOI:** 10.3389/fmolb.2025.1519977

**Published:** 2025-03-12

**Authors:** Weipu Mao, Tracy Zhang, Huan Chen, Sagar Barge, Zongwei Wang, Aria Olumi, Seth Alper, Weiqun Yu

**Affiliations:** ^1^ Department of Medicine, Beth Israel Deaconess Medical Center and Harvard Medical School, Boston, MA, United States; ^2^ Department of Urology, Beth Israel Deaconess Medical Center and Harvard Medical School, Boston, MA, United States

**Keywords:** activin signaling, follistatin, urothelium, epithelial-interstitial cell interaction, bladder cancer

## Abstract

The activin-follistatin-inhibin (AFI) axis plays a crucial role in sexual development and reproduction. Recently it was demonstrated that these proteins are also synthesized by many local tissues and regulate different biological activities, including tissue regeneration and cancer metastasis. However, little is known about the expression profile of the AFI axis in the bladder and its role in bladder function and dysfunction. We have examined the expression profile of 11 AFI family members in the mouse bladder. INHA, INHBA, and follistatin are the major ligand subunits detected among the six examined in the bladder. ACVR1, ACVR1B, and ACVR2B are the major receptor subunits detected among the five examined in the bladder. Immunolocalization studies reveal unique cellular distributions of these ligands and receptors within the bladder. The urothelial-localized ACVR2B/ACVR1B receptor complex suggests a role of activin signaling in urothelial function. The stimulatory activin A is present only in a subset of interstitial cells, separated from the urothelial activin receptor ACVR2B/ACVR1B by a basement membrane containing accumulated inhibitory ligand FST and by a layer of activin-negative myofibroblasts. This spatial information on AFI signal molecules suggests that activin A-positive interstitial cells might regulate urothelial cell function via paracrine signaling through activin A-ACVR2B/ACVR1B interaction. Further analysis of the human bladder confirmed the expression profile of the AFI axis, and revealed significantly upregulated expression of INHBA–ACVR2B in bladder cancer. These data suggest roles for these molecules in the growth and metastasis of bladder cancer, and highlight their potential as diagnostic and prognostic biomarkers.

## Introduction

Activin (ACT), inhibin (INH), and follistatin (FST) were initially isolated in the mid-1980s from the follicle fluids of humans, pigs, rats, and other mammals as follicle-stimulating hormone (FSH) modulators (Welt et al., 2002; Tumurgan et al., 2019; Fullerton et al., 2017). ACT was discovered to stimulate the pituitary gland to release FSH, while INH and FST inhibited FSH release. FSH is central to sexual development and reproduction, signaling the ovaries in females to produce estrogen and control the ovulatory and menstrual cycles, while signaling the testes in males to produce testosterone and regulate spermatogenesis (Lispi et al., 2023). The activin-follistatin-inhibin (AFI) axis is thus an important endocrine hormone system for animal reproductive functions (Bouzoni et al., 2020). Although ACT, FST, and INH are secreted at the highest levels by pituitary cells and gonads to regulate reproduction, subsequent studies have demonstrated that these proteins of the AFI axis are also synthesized by many nonreproductive tissues and regulate numerous additional biological activities (Namwanje and Brown, 2016; Wijayarathna and Hedger, 2019; Seachrist and Keri, 2019).

Activins are dimers formed by βA or βB subunits as either heteromers (βAβB) or homomers (βAβA, or βBβB), while activin βA or βB subunits dimerization with the inhibin α subunit forms the inhibins (αβA, αβB) (Namwanje and Brown, 2016). Activins bind to activin receptors type IIA (ACVR2A) and IIB (ACVR2B) to activate serine-threonine kinase activity. Activin type II receptor autophosphorylation recruits type I activin receptors to form a functional receptor complex. The activated receptor complex initiates SMAD-signaling by phosphorylating the SMAD 2/3/4 complex, enabling its nuclear translocation followed by transcriptional modulation of target genes (Barrero et al., 2023). The less well-understood activins βC and βE are believed to serve as antagonists (Namwanje and Brown, 2016). Inhibin binding to activin receptors also antagonizes activin signaling, while FST directly binds to activins to promote their endocytosis and lysosomal degradation. Beyond regulation of reproduction, activin signaling is increasingly recognized as important in modulating development, aging, metabolism, tissue homeostasis, wound healing, fibrosis, cancer, and inflammation (Bouzoni et al., 2020).

Within the urinary system, the AFI axis has been proposed to contribute to prostate morphogenesis, cell apoptosis, and tissue homeostasis (Gold et al., 2017). mRNAs encoding the activin βA subunit and the type II activin receptors have been detected in the rat urinary bladder (Ying and Zhang, 1995), and increased bladder FST levels were observed in a mouse model of cyclophosphamide-induced cystitis (Shaffer et al., 2017). Increased INHβA mRNA levels have also been reported in human bladder cancer (Lee et al., 2015; Kao et al., 2022). These preliminary reports suggest possible roles of activin signaling in urinary bladder physiology and pathology. Thus, a complete survey of the bladder wall expression and localization of ACT and related proteins comprising the AFI axis should provide valuable information for further mechanistic understanding of the AFI axis in bladder function and dysfunction.

## Material and methods

### Materials

Unless otherwise specified, all chemicals were obtained from Sigma (St. Louis, MO) and were of reagent grade or better.

### Animals

Both male and female C57BL/6J mice used in this study were aged between 12 and 16 weeks and were purchased from Jackson Laboratory (Bar Harbor, ME). Mice were housed in standard polycarbonate cages, maintained on a fixed 12-h light, 12-h dark cycle at 25°C, and had free access to regular food and water. Mice were euthanized by inhalation of 100% CO_2_, followed by the swift removal of the bladder. Subsequent processing of the bladder was carried out according to the described method. All animal studies were conducted in accordance with the National Institutes of Health guidelines for animal care and use and were approved by the Animal Care and Use Committee of Beth Israel Deaconess Medical Center under protocol #010-2022.

### Quantitative RT- PCR

A fresh mouse bladder was homogenized in a glass tissue grinder, and 1 mL of TRIzol reagent (Invitrogen, United States) was used to extract the total RNA according to the previous experimental procedure (Xie et al., 2021). The quality and quantity of the extracted total RNA were then assessed by a Nanodrop 2000 spectrophotometer (Thermo Fisher Scientific, United States). After cDNA synthesis by the reverse transcription of 1 μg total RNA using the SuperScript IV First-Strand Synthesis System (#18091050, Invitrogen, United States), the gene expression levels in the bladder were subsequently quantitated using the Maxima SYBR Green/ROX qPCR Master Mix kit (#K0222, Thermo Fisher Scientific, United States) in the Applied Biosystems 7300 real-time PCR system (Thermo Fisher Scientific, United States). Thermal cycling conditions were as follows: UDG pre-treatment at 50°C for 2 min, initial denaturation at 95°C for 10 min, followed by 40 cycles of denaturation at 95°C for 15 s and annealing/extension at 60°C for 60 s. The relative expression of individual genes was calculated using the 2^−ΔΔCt^ method, with SDHA (succinate dehydrogenase complex, subunit A) serving as the internal control. To compare the expression levels between males and females, and also among different genes, we further chose the highest expressed gene as 100%. Primer details are available in Table 1. The melting curves for each gene’s primers are available in Supplementary Figure 2.

**TABLE 1:** Primers used for RT-qPCR.

AFI	Sequence of primers (5′-3′)
Acvr1	CCATTGAAGGGCTCATCACCAC
	CCGTTCTCTGTACCAGGAAAGG
Acvr1b	ACGAAGATGCAATTCTGGAGG
	TCTTTCCCATCACTCGCAAG
Acvr1c	GCTGACATCTATTCGGTGGG
	TTTGGGAGATTTGGTCGGAG
Acvr2a	GCGACATTGTTTTGCTACCTG
	ACACATATTGCCCTCACAGC
Acvr2b	AAGCCTTCTATTGCCCACAG
	TCAAACCGAACAGCCAGG
Fst	TGTAATCGGATTTGCCCAGAG
	CACACTGGATATCTTCACAGGAC
Inha	CTAGACAGAAAGGGCACAGG
	AGGGTCAACAGCAAAAGGAG
Inhba	ATCACCTTTGCCGAGTCAG
	TGCTGAAATAGACGGATGGTG
Inhbb	TCCGAGATCATCAGCTTTGC
	GGGAGCAGTTTCAGGTACAG
Inhbc	TGACAGGGACAGCAACATTG
	GGACAGAAGTGGGAACAGAG
Inhbe	CTGCTTCTGTATCCTCTTTGGG
	CTTCTACTCTGCACCCACAC

### Western blot analysis

Proteins were extracted using 500 ul RIPA buffer (150 mM NaCl, 50 mM Tris, 1% v/v NP-40, 0.5% deoxycholic acid, and 0.1% w/v SDS, pH 7.4) containing protease inhibitors (Roche Applied Science, United States) on ice. The protein concentration was determined using the Pierce BCA Protein Assay Kit (#WF325489, Thermo Fisher Scientific, United States). Denatured protein species (50 μg per well) were separated in 8%–16% 12-well Tris-Glycine precast gels (NB12-816, NuSeq, United States) according to standard protocols and were then blotted to polyvinylidene fluoride membranes (#ISEQ00010, Sigma-Aldrich, United States). The membranes were blocked with 5% skimmed milk for 1 h and then incubated overnight at 4°C with the primary antibody, followed by incubation with the corresponding secondary antibody at room temperature for 1 h. After three washes with TBS containing 0.1% Tween 20, the membrane was exposed to the Western Lightning Plus-ECL reagent (#203-22111, PerkinElmer, Netherlands) and scanned with an ArtixScan 1800f flatbed scanner (Microtek International, Carson, CA). β-actin (1:1000, #MA1-744, Invitrogen, United States) was used as an internal control, and contrast was corrected using Photoshop (San Jose, United States). The relative intensity of protein bands was quantitated by Fiji software. Restore Western Blot Stripping Buffer ((#sc-281698, Santa Cruz, United States) was used to elute antibodies for repeated staining. The protein expression levels are compared between males and females with the high expressed one set as 1. Antibodies details are available in Table 2.

**TABLE 2:** Antibody list.

Antibody name	Manufacturer	Catalog number	Application, dilution
Inha	ABclonal	A1734	WB, 1:1000
Inha	BIOSS	Bs-1032R	IF, 1:100
Inhba	R&D system	AF338	WB, 1:1000; IF, 1:100
Inhbe	BIOSS	Bs-16658R	WB, 1:1000; IF, 1:100
Fst	R&D system	AF669	WB, 1:1000; IF, 1:100
Acvr1	ABclonal	A19274	WB, 1:1000
Acvr1	Proteintech	67417-1-Ig	IF, 1:100
Acvr1b	ABclonal	A21140	WB, 1:1000
Acvr1b	BIOSS	Bs-6018R	IF, 1:100
Acvr2b	BIOSS	Bs-12417R	WB, 1:1000
Acvr2b	Invitrogen	PA5-28231	IF, 1:100
β-actin	Invitrogen	MA1-744	WB, 1:1000
Entpd3	R&D system	AF4464	IF, 1:100
Tenascin C	R&D system	3358-TC-050	IF, 1:100
Alpl	R&D system	AF2910	IF, 1:100
CD34	Abcam	ab8158	IF, 1:100
Krt5	Biolegend	905,901	IF, 1:100

### Immunofluorescence analysis

Freshly excised bladders were fixed in 4% wt/vol paraformaldehyde (PFA) dissolved in 100 mM sodium cacodylate (pH 7.4) buffer for 2 h at room temperature. The fixed bladders were preserved in tissue cassettes using an optimal cutting temperature (OCT) compound. Tissue was then sectioned (5 µM), and incubated overnight at 4°C with primary antibody (1:100), followed by incubation with corresponding dye-conjugated secondary antibody (1:100 dilution). Nuclei were stained with DAPI. Imaging was performed on a BX60 Olympus fluorescence microscope with cellSens v4.3 software using a 40×/0.75 magnification objective. Images (512 & 512 pixels) were saved as TIFF files. Fluorescent signals taken with different wavelengths were merged into one image using Adobe Photoshop, and the contrast level of the final image was adjusted. Antibodies details are available in Table 2.

### Gene expression of AFI axis components in human bladder cancer

Gene expression analysis of AFI axis components in human bladder cancer tissue was performed using The Cancer Genomics Atlas (TCGA) database. In RNA-seq data for 433 bladder cancer cases from the TCGA database, we found 19 cases including data from both bladder cancer and from adjacent “normal” bladder tissue as matched controls. The data from these 19 cases (10 males and 9 females) were analyzed in TPM (Transcripts Per Million) format. Complete gene expression profiles of the bladder cancer cases were downloaded using The Genomic Data Commons (GDC) Data Transfer Tool (https://gdc.cancer.gov/access-data/gdc-data-transfer-tool).

### Data analysis

GraphPad Prism 8.3 software (San Diego, United States) was used for statistical analyses. All data are expressed as means ± SD, or presented as boxes and whiskers (extending from minimum to maximum values). Data were analyzed by two-tail Student’s t-test between two groups. P < 0.05 was considered significant.

## Results

### Messenger RNA (mRNA) expression of AFI axis family genes in the mouse bladder

**FIGURE 1:**
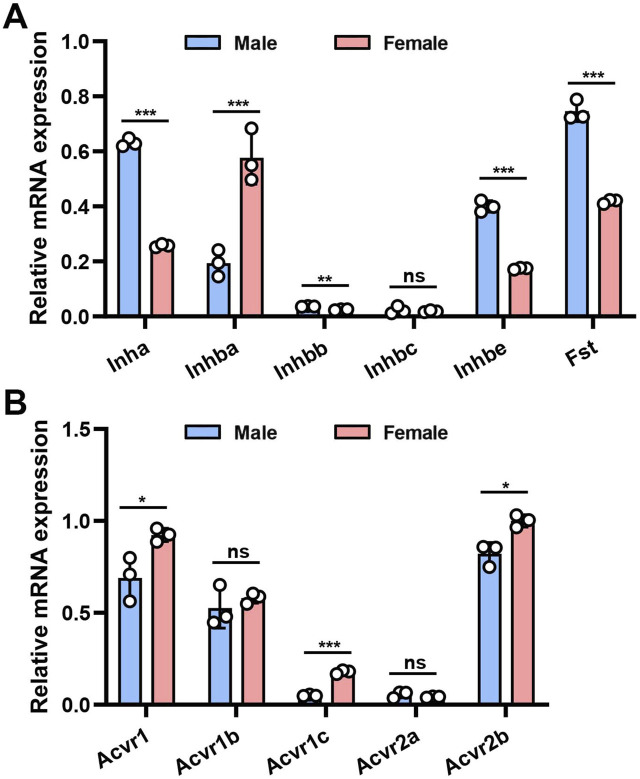
mRNA expression of AFI axis family members in the bladder of male and female mice. **(A)** Differences in mRNA expression of *Inha, Inhba, Inhbb, Inhbc, Inhbe,* and *Fst* in the bladder of male and female mice. **(B)** Differences in mRNA expression of *Acvr1, Acvr1b, Acvr1c, Acvr2a,* and *Acvr2b* in the bladder of male and female mice (n = 3, Data were analyzed with the use of Student’s t-test, **p < 0.01, ***p < 0.001).

We initially applied quantitative RT-PCR (qRT-PCR) to investigate the mouse bladder mRNA expression profiles of 11 genes encoding the AFI axis family members. These genes included *Inha*, *Inhba*, *Inhbb*, *Inhbc*, *Inhbe*, and *Fst* encoding AFI axis ligands, and *Acvr1*, *Acvr1b*, *Acvr1c*, *Acvr2a*, *Acvr2b* encoding AFI axis receptors. Our data revealed in both male and female bladders abundant expression of mRNAs encoding the four ligand subunit genes *Inha*, *Inhba*, *Inhbe*, and *Fst*, and the three receptor subunit genes *Acvr1*, *Acvr1b*, and *Acvr2b* (Figure 1). The mRNA expression levels of other ligand and receptor subunits were lower or even undetectable (Figure 1A). We then compared gene expression levels between males and females. Ligand subunit genes *Inha*, *Inhbe*, and *Fst* exhibited higher expression in male than in female mouse bladder, whereas *Inhba* was expressed at higher levels in female than in male mouse bladder. The major receptor genes *Acvr2b* and *Acvr1* were also expressed at higher levels in female than in male bladder (Figure 1A). The data suggest that female bladders might exhibit higher levels of activin signaling than male bladders, but the mechanism and physiological importance of this sexually dimorphic expression pattern remains unclear.

### Western blot analysis confirms bladder expression of AFI axis family members

**FIGURE 2:**
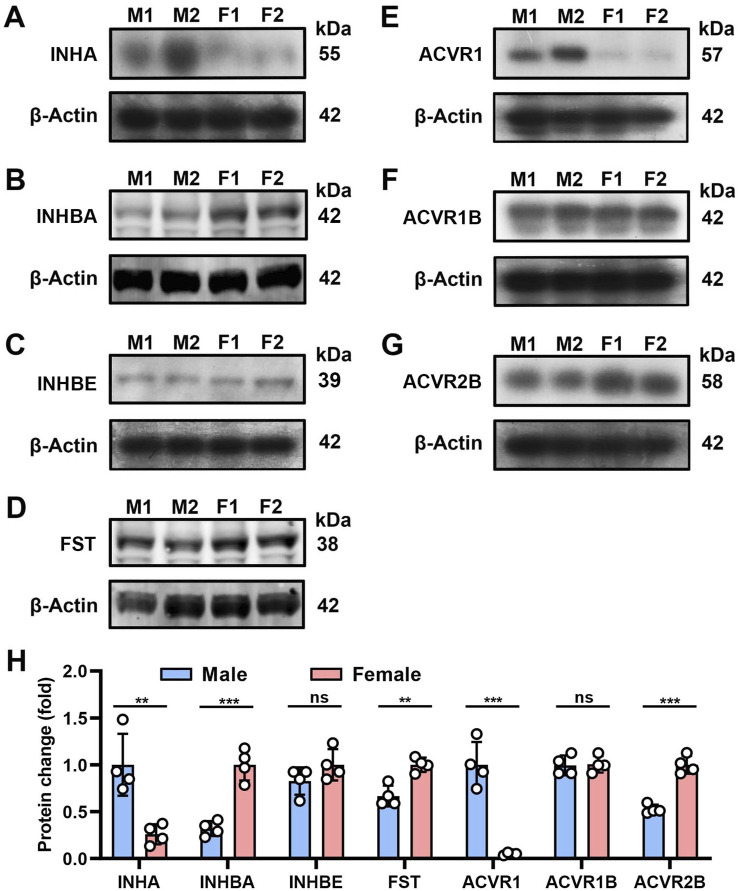
Protein expression of AFI axis family members in the bladder of male and female mice. **(A–G)** Western blot analysis of INHA **(A)**, INHBA **(B)**, INHBE **(C)**, FST **(D)**, ACVR1 **(E)**, ACVR1B **(F)**, ACVR2B **(G)** in both male and female mouse bladder (25 μg protein loaded). **(H)** Differences in protein expression of AFI axis family members in the bladder of male and female mice. (n = 4, molecular values of major detected protein species are indicated at the right of each panel. Data were analyzed with the use of a two-tailed Student’s t-test, **p < 0.01, ***p < 0.001).

We selected seven highly expressed AFI members for further Western blot analysis. As shown in Figure 2, specific protein bands were detected for the ligand subunits INHA, INHBA, INHBE and FST, with molecular values of 55, 42, 39, and 38 kDa. For AFI receptor subunits ACVR1, ACVR1B, and ACVR2B, specific protein bands with molecular values of 57, 42, and 58 kDa were detected (Figures 2A–G). The observed differences in male and female protein expression (Figure 2H) were generally consistent with the above-noted mRNA expression profiles.

### AFI axis family members are expressed in urothelial cells and in a subset of interstitial cells

**FIGURE 3:**
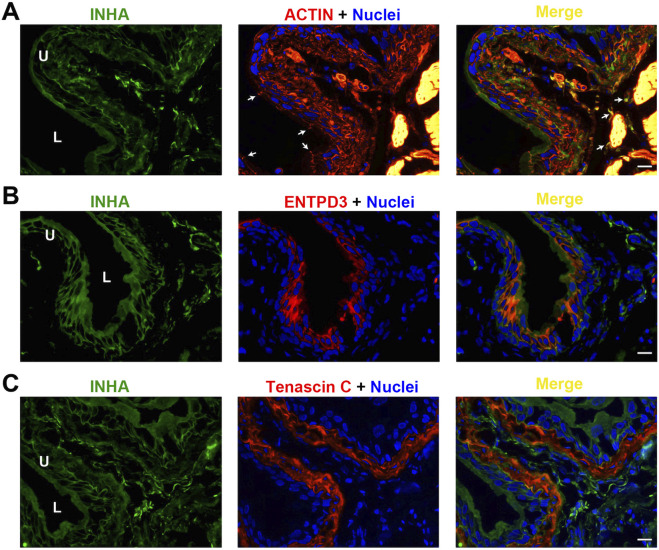
Localization of INHA in mouse bladder. Cryosections of mouse bladder tissue were labeled with INHA antibody (green) and rhodamine phalloidin **(A)**, or antibodies to ENTPD3 **(B)** or Tenascin C **(C)**; all displayed in red. Nuclei were labeled with DAPI (blue). *Far right*: merged images. Arrowheads indicate tight junctions of umbrella cells. L, bladder lumen. U, urothelium. Scale bars = 10 μm.

We performed immunofluorescence microscopy and imaging to understand the cellular distribution of these AFI ligands and receptors in the bladder wall. As shown in Figure 3, INHA is localized in both urothelial cells and in the interstitial compartment. Urothelial cell INHA predominantly colocalized with the cell membrane, counterstained with rhodamine-phalloidin to visualize the membrane actin cortex (Figure 3A). INHA was also colocalized with ENTPD3 (Figure 3B), an ATP-converting enzyme expressed in the urothelial cell basolateral membrane. INHA was also expressed in a subset of interstitial cells distributed throughout the lamina propria and surrounding the smooth muscle bundles. Interestingly, the INHA-positive interstitial cells were excluded from the tenascin C-positive layer of lamina propria adjacent to the urothelial cells. These interstitial cells also expressed the myofibroblast marker αSMA (Yu et al., 2012). We thus concluded that INHA is expressed in non-myofibroblast interstitial cells of the bladder wall (Figure 3C).

**FIGURE 4:**
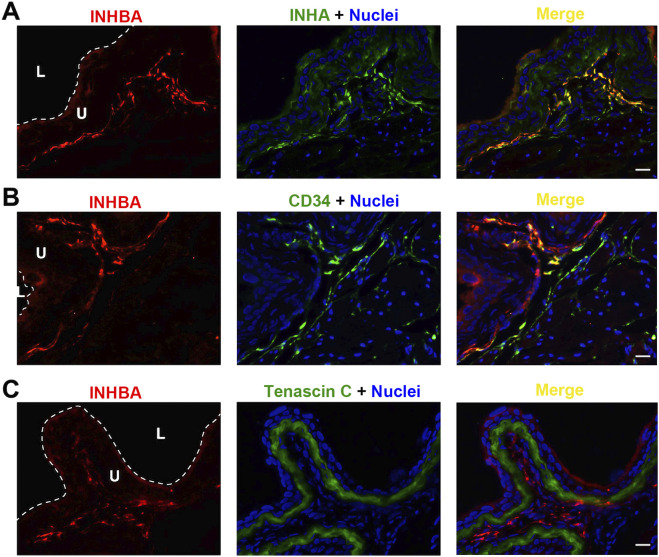
Localization of INHBA in mouse bladder. Cryosections of mouse bladder tissue were labeled with INHBA antibody (red) and INHA **(A)**, or antibodies to CD34 **(B)**, or Tenascin C **(C)**; all displayed in green. Nuclei were labeled with DAPI (blue). *Far right*: merged images. L, bladder lumen. U, urothelium. Scale bars = 10 μm.

INHBA is predominantly detected in the lamina propria, where it colocalizes with INHA (Figure 4A). INHBA also seems to be expressed in interstitial cells dispersed among the smooth muscle bundles, but with relatively weaker intensity. This expression profile is also evident in INHBA co-stained with CD34, another biomarker for bladder wall interstitial cells (Figure 4B). Same as for INHA, the INHBA-positive cells are not tenascin C-positive, and thus not myofibroblasts (Figure 4C). We could not find specific and immunolocalization-competent antibodies that recognize INHBE.

**FIGURE 5:**
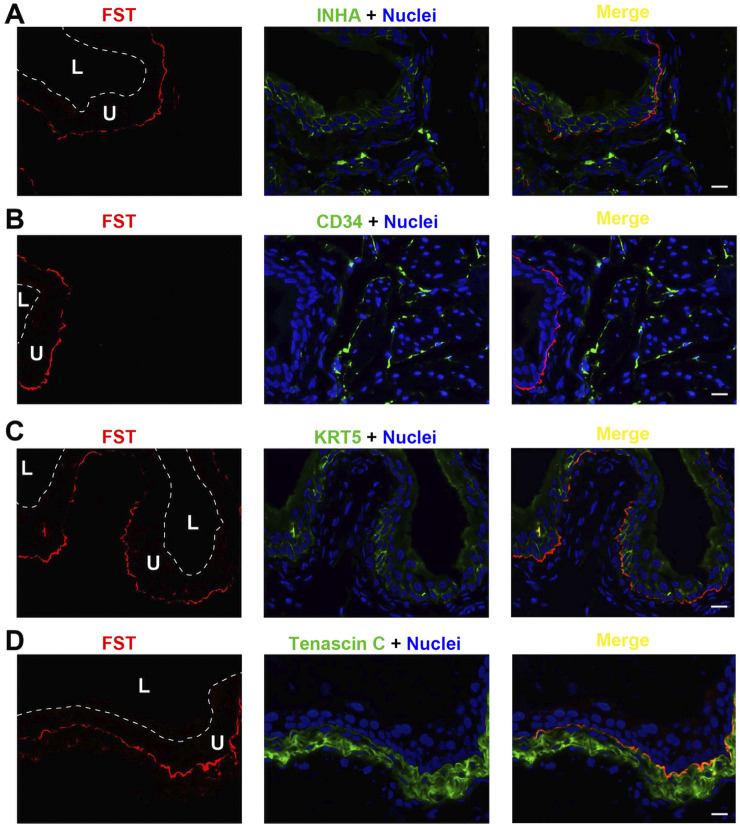
Localization of FST in mouse bladder. Cryosections of mouse bladder tissue were labeled with FST antibody (red) and INHA **(A)**, or antibodies to CD34 **(B)**, or KRT5 **(C)** or Tenascin C **(D)**; all displayed in green. Nuclei were labeled with DAPI (blue). *Far right*: merged images. L, bladder lumen. U, urothelium. Scale bars = 10 μm.

FST exhibited a unique expression profile, shown in Figure 5. FST colocalized neither with INHA in urothelial cells (Figure 5A) nor with the interstitial cell biomarker CD34 (Figure 5B). Interestingly, FST was detected immediately subjacent to the basal, KRT5-positive urothelial cells, (Figure 5C). FST partially colocalized with tenascin C as well (Figure 5D). Thus, FST appears to be found in a unique thin basement membrane-like structure between the urothelial cells and the underlying myofibroblasts.

**FIGURE 6:**
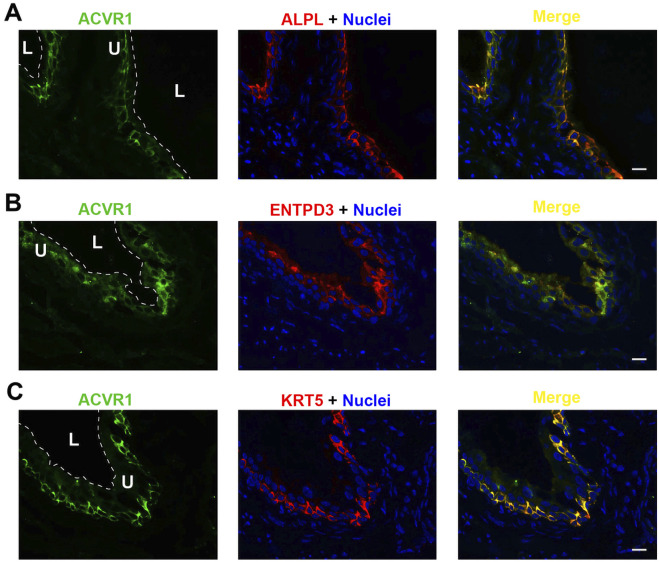
Localization of ACVR1 in mouse bladder. Cryosections of mouse bladder tissue were labeled with ACVR1 antibody (green) and ALPL **(A)**, or antibodies to ENTPD3 **(B)** or KRT5 **(C)**; all displayed in red. Nuclei were labeled with DAPI (blue). *Far right*: merged images. L, bladder lumen. U, urothelium. Scale bars = 10 μm.

**FIGURE 7:**
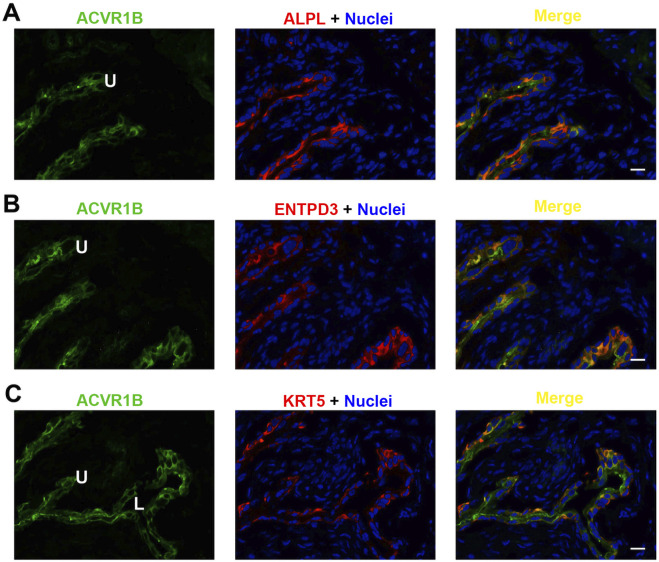
Localization of ACVR1B in mouse bladder. Cryosections of mouse bladder tissue were labeled with ACVR1B antibody (green) and ALPL **(A)**, or antibodies to ENTPD3 **(B)** or KRT5 **(C)**; all displayed in red. Nuclei were labeled with DAPI (blue). *Far right*: merged images. L, bladder lumen. U, urothelium. Scale bars = 10 μm.

**FIGURE 8:**
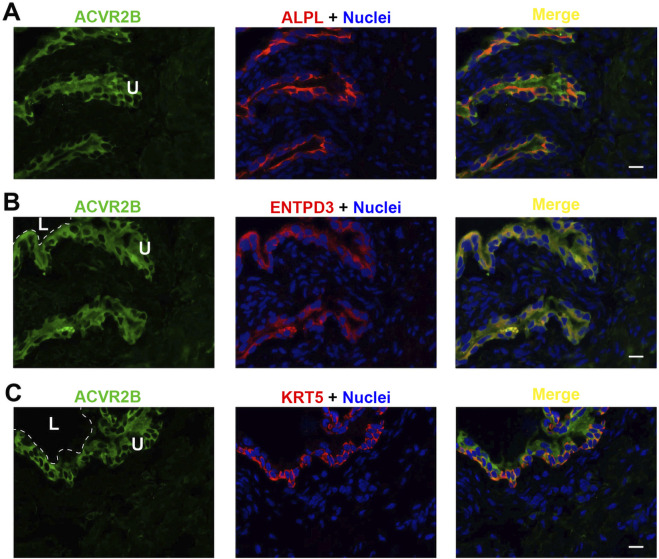
Localization of ACVR2B in mouse bladder. Cryosections of mouse bladder tissue were labeled with ACVR2B antibody (green) and ALPL **(A)**, or antibodies to ENTPD3 **(B)** or KRT5 **(C)**; all displayed in red. Nuclei were labeled with DAPI (blue). *Far right*: merged images. L, bladder lumen. U, urothelium. Scale bars = 10 μm.

Each of the three receptor subunits ACVR1, ACVR1B, and ACVR2B exhibited similar staining patterns in urothelial cells, as shown in Figures 6–8, but not in other bladder wall cell types. ACVR1, ACVR1B, and ACVR2B colocalized with the urothelial basolateral membrane proteins ENTPD3 and ALPL (alkaline phosphatase) (Figures 6A, B, 7A) (Yu, 2015; Yu et al., 2011). They also colocalized with KRT5 in urothelial basal cells (Figures 6C, 7C, 8C). Thus, the activin/inhibin receptors of the bladder are expressed predominantly in urothelial basolateral membranes.

### AFI axis family members exhibit altered expression profiles in human bladder cancer

**FIGURE 9:**
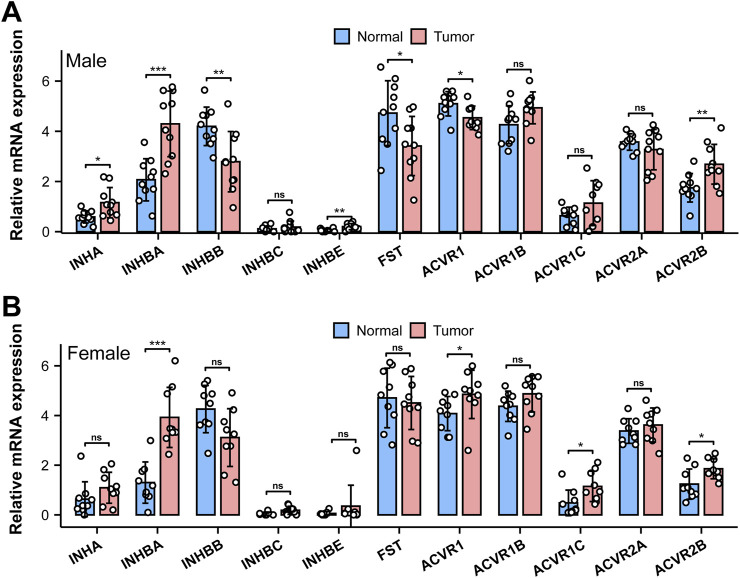
mRNA expression of AFI axis family members in human bladder cancer. Differences in mRNA expression of AFI axis components in male [**(A)** n = 10] and female [**(B)**, n = 9] bladder cancer compared to the adjacent “normal” tissue. (Data were analyzed with the use of a two-tailed Student’s t-test, **p < 0.01, ***p < 0.001).

The AFI axis has been proposed to play an important role in the growth and metastasis of bladder cancer (Lee et al., 2015; Kao et al., 2022). We therefore analyzed gene expression profiles of AFI axis components in the TCGA database. As compared to the AFI axis expression profile in mouse bladder, *INHBB* and *ACVR2A* were highly expressed in human bladder, whereas expression of *Inhbb* and *Acvr2a* in mouse bladder was near the detection threshold (Figures 1, 9). Of particular interest, expression of INHBA and ACVR2B were increased in bladder cancer in both males and females, as compared to adjacent normal bladder tissue (Figure 9).

## Discussion

The proteins of the AFI axis are part of the TGF-β superfamily, and the AFI axis plays an important role in regulating reproductive function, tissue development, insulin sensitivity (energy supply), muscle wasting, and osteoporosis (Bouzoni et al., 2022; Perakakis et al., 2019; Perakakis et al., 2018a; Perakakis et al., 2018b). Although initially discovered in the ovary, pituitary, and gonads, later studies indicated a wide distribution of the AFI axis in many organs. The role of the AFI axis in bladder physiology has previously been little investigated. However, our qRT-PCR and Western blot data revealed high expression of 7 gene products in both male and female mouse bladders (Figures 1, 2), suggesting a potential role of the AFI axis in bladder tissue homeostasis. Analysis of the TCGA database also revealed the expression of 9 AFI genes in male and female human bladders (Figure 9), confirming the relevance of the AFI axis in human bladder function.

INHA is the essential subunit of dimeric inhibin ligands containing different β subunits (Namwanje and Brown, 2016), and our qRT-PCR and Western blot analyses each detected INHA expression in mouse bladder. However, among the 4 β subunits we examined, only INHBA and INHBE were detected. These data suggest that INHA and INHBA could dimerize to form Inhibin A, and INHBA homodimers could form activin A in the bladder wall. INHBE might also be able to dimerize with INHA and INHBA, but information on the dimerization and function of this subunit is limited.

INHA was also detected in the human bladder, but at apparently lower levels of expression than in mouse bladder, and at lower expression levels than those of INHBA, FST, and other AFI genes (Figures 1, 9). Although INHBA was highly expressed in both human and mouse bladders, INHBB was detected at levels comparable to that of INHBA in the human bladder, whereas INHBA was expressed at a low level in the mouse bladder. The combined presence of INHA, INHBA, and INHBB in the human bladder suggests a potentially complex protomer dimerization pattern yielding activin A, activin B, activin AB, inhibin A, and inhibin B (Namwanje and Brown, 2016). Although the dominant (dimeric) activins and inhibins of the human bladder require further investigation, the increased expression of INHBA and decreased INHBB expression together suggests that activin A might be a major agonist stimulating bladder cancer growth and metastasis, and thus could serve as a biomarker for bladder cancer diagnosis and prognosis (Lee et al., 2015; Kao et al., 2022).

Activin binds to receptors ACVR2A or ACVR2B to initiate cellular signaling, and ACVR2B is a major subunit in the mouse bladder wall. Upon binding, ACVR2B can phosphorylate ACVR1B or ACVR1C receptors to form a receptor complex. As ACVR1B is the dominant subunit in the mouse bladder, ACVR2B/ACVR1B may constitute the principal functional receptor complex in the mouse bladder wall. ACVR1 is activated by bone morphogenic proteins (BMPs) (Olsen et al., 2015), which are also expressed in the bladder. The ability of activin ligation of ACVR2B to recruit ACVR1 in the bladder wall remains unclear.

In addition to ACVR2B, ACVR2A was also significantly expressed in the human bladder, suggesting potentially complex functional roles for AFI signaling in the human bladder wall. It is noteworthy that ACVR2B expression increased significantly in human bladder cancers of both males and females, whereas ACVR2A expression was unchanged (Figure 9). This pattern suggests the potential involvement of activin A ligand binding to the ACVR2B receptor in promoting bladder cancer growth and metastasis.

**FIGURE 10:**
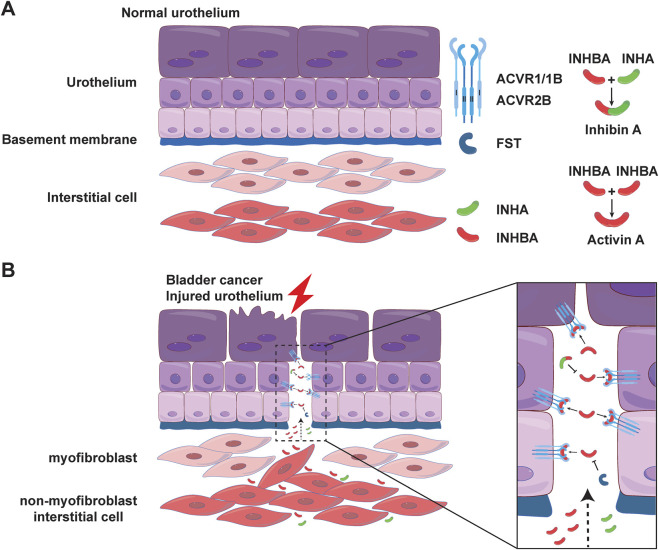
Location and possible mode of action of AFI family members in the bladder. Possible modes of action of 7 family members in the normal **(A)** and injured **(B)** uroepithelium. In normal urothelium, a layer of myofibroblasts under the FST barrier further separates activin A from urothelial cells. In injured urothelium, the FST and myofibroblast barriers may be disrupted, allowing activin A to cross the barrier and act as a paracrine signal to activate the ACVR2B/ACVR1B receptor complex in urothelium.

Our morphological study on cellular localization in the mouse bladder further implies a potential signaling mechanism in the bladder wall. As shown in Figures 6–8, ACVR2B/ACVR1B receptor complex is only present in urothelial cells, but not in other types of cells, suggesting its importance in modulating urothelial function. However, the major stimulatory ligand activin A is detected only in the non-myofibroblast interstitial cells. Interestingly, the inhibitory subunit INHA is also present in the urothelial cell layer, and colocalizes with activin A signaling in interstitial cells as well. Moreover, the inhibitory FST ligand is found in a thin basement membrane-like barrier between the urothelial ACVR2B/ACVR1B receptor complex and the stimulatory ligand activin A in bladder interstitial cells. This FST barrier is likely synthesized by a subset of myofibroblasts or fibroblasts in the lamina propria, and high levels of FST mRNA expression have been demonstrated in these cells by single-cell RNA sequencing of both human and mouse bladders (Yu et al., 2019). Activin A is further separated from the urothelial cells by the myofibroblast cell layer beneath the FST barrier. This spatial distribution of receptors and ligands seems to provide no accessible stimulatory ligands for the ACVR2B/ACVR1B receptor complex (Figure 10A). This situation might reflect normal conditions of urothelial cell quiescence with rare cell proliferation. However, in pathological conditions such as tissue injury or cancer, this FST and myofibroblast barrier might be disrupted, allowing activin A to cross the FST barrier and activate the ACVR2B/ACVR1B receptor complex in urothelial cells as a paracrine signal (Figure 10B). Activin A-positive interstitial cells in this scenario might also migrate across the FST barrier and form scar tissue.

The cellular localizations of AFI axis components in the human bladder require further investigation. However, the predominantly upregulated INHBA and ACVR2B in the human bladder are fully consistent with the major AFI components and their orthologs in the mouse bladder, suggesting the importance of these particular AFI molecules and potentially similar localization and functional similarity. Elevated stromal activin A release from substrates of increasing stiffness has been shown to promote ligand-dependent epithelial cell migration and epithelial-to-mesenchymal transition (EMT) in the context of carcinogenesis (Bauer et al., 2020). In addition to INHBA, reported as the most upregulated gene in human bladder cancer, we also observed upregulation of ACVR2B in bladder cancer. Knockdown of INHBA inhibited bladder cancer cell proliferation and migration in culture (Kao et al., 2022). Thus, the unique spatial distribution of the ACVR2B/ACVR1B receptors, the isolated stimulatory activin A ligand, and the inhibitory inhibin A and FST barrier appear tightly regulated for tissue homeostasis (Figure 10). Future research endeavors should focus on the mechanistic understanding of AFI components, their interactions, and their involvement in the normal, diseased, and malignantly transformed bladder tissue.

In summary, we have identified the AFI axis members in the bladder wall and determined their cellular localizations. We further proposed a working model for the potential mechanism of AFI signaling across multiple bladder tissue layers, in which interstitial cells’ paracrine agonist activins signal urothelial cells for possible cell regeneration or tumorigenesis.

## Data Availability

The data presented in the study are deposited in the Harvard Dataverse repository: https://dataverse.harvard.edu/dataset.xhtml?persistentId=doi:10.7910/DVN/DRUJLQ.
