# Supplementary figures and images for: Expression and distribution of activin-follistatin-inhibin axis in the urinary bladder
Source: Front Mol Biosci. 2025 Mar 12;12:1519977. doi: 10.3389/fmolb.2025.1519977 (PMC11936821; doi:10.3389/fmolb.2025.1519977)

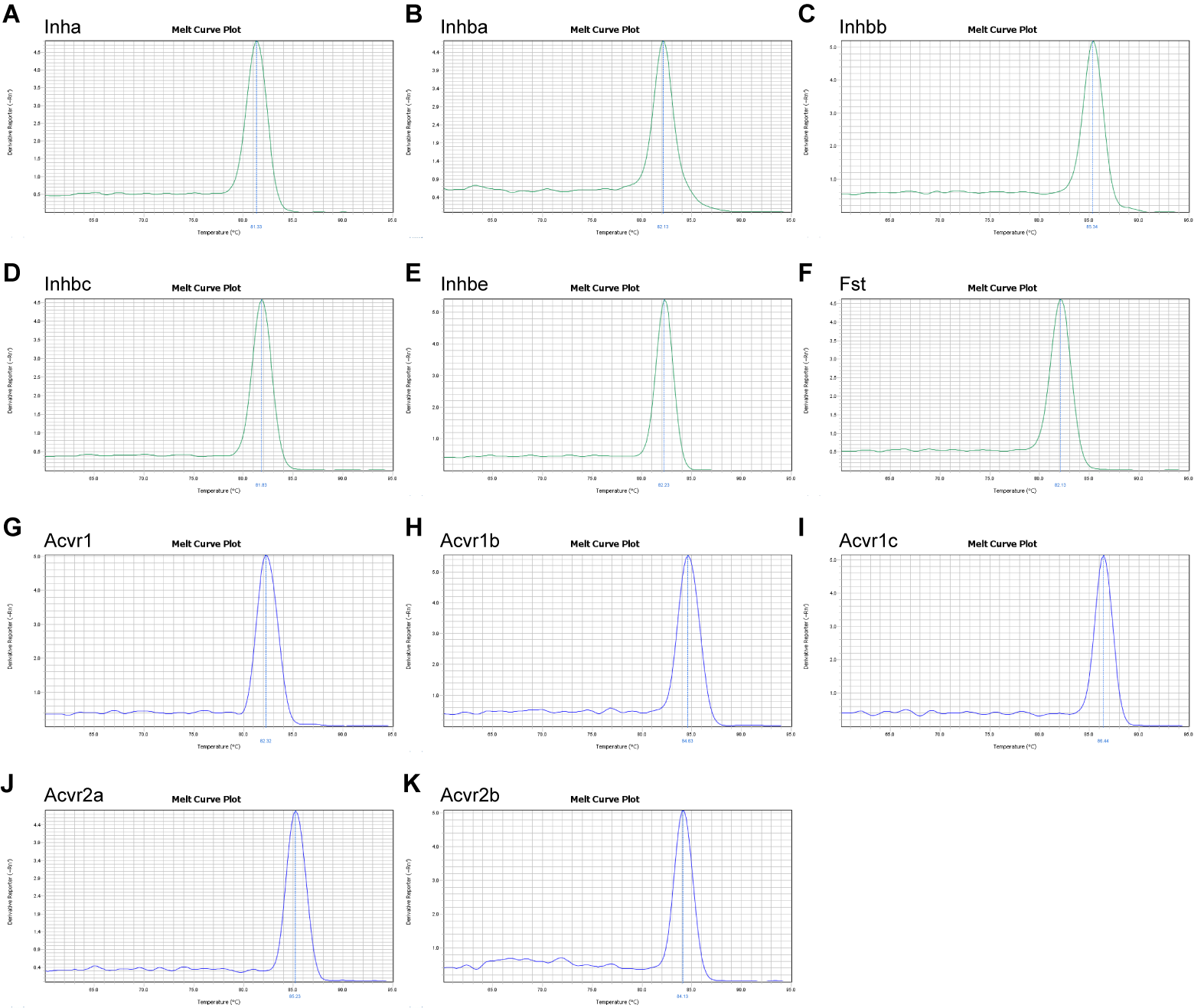

Supplement: Supplementary file 1 [file Image2.tif]

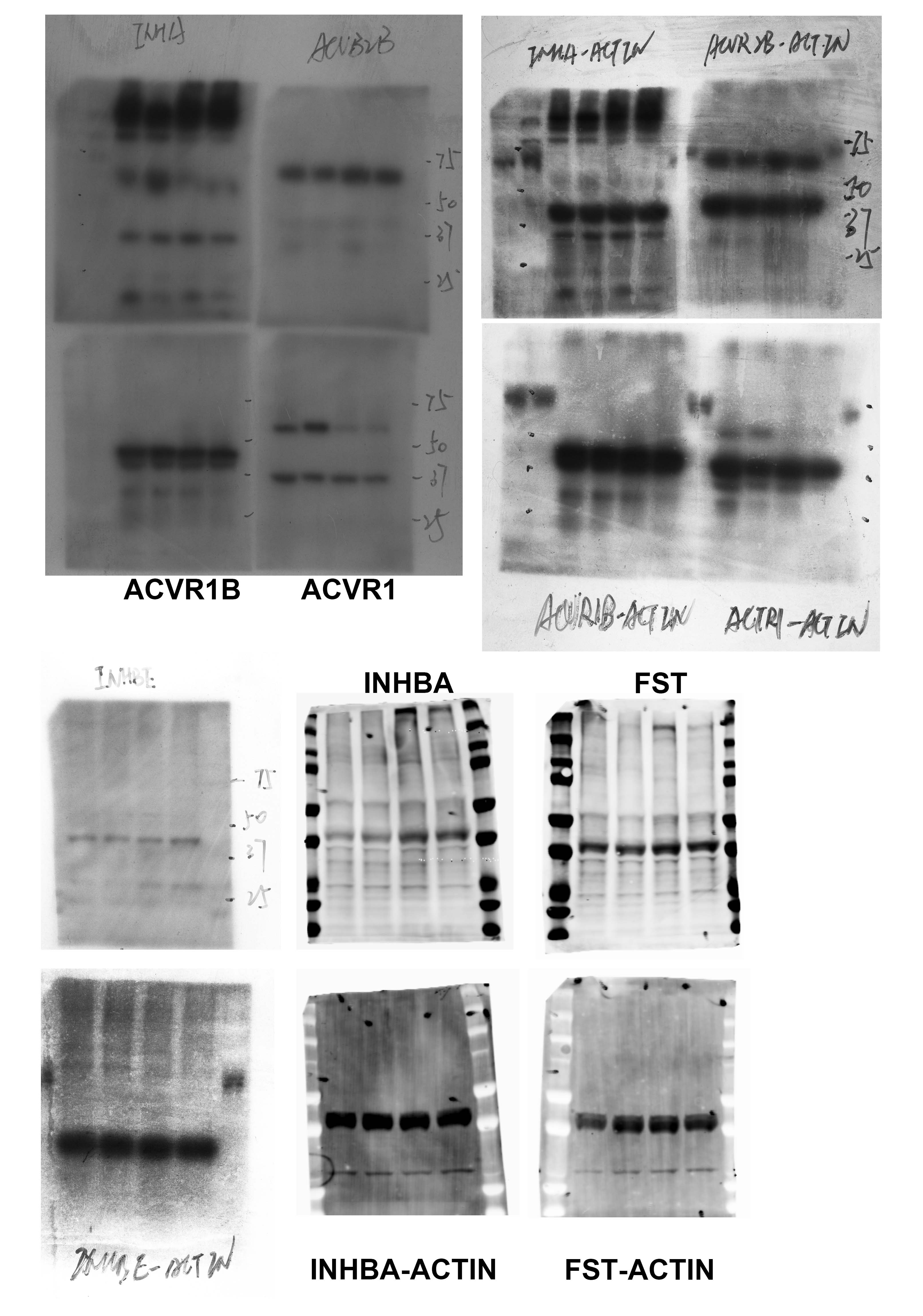

Supplement: Supplementary file 2 [file Image1.tif]
